# Stratification of reactivity determines nitrate removal in groundwater

**DOI:** 10.1073/pnas.1816892116

**Published:** 2019-01-28

**Authors:** Tamara Kolbe, Jean-Raynald de Dreuzy, Benjamin W. Abbott, Luc Aquilina, Tristan Babey, Christopher T. Green, Jan H. Fleckenstein, Thierry Labasque, Anniet M. Laverman, Jean Marçais, Stefan Peiffer, Zahra Thomas, Gilles Pinay

**Affiliations:** ^a^Centre National de la Recherche Scientifique (CNRS), Géoscience Rennes - UMR 6118, Université de Rennes, 35042 Rennes, France;; ^b^Centre National de la Recherche Scientifique (CNRS), Institut National de la Recherche Agronomique (INRA), Observatoire des Sciences de l’Univers de Rennes (OSUR) - UMR 3343, Université de Rennes, 35042 Rennes, France;; ^c^Department of Plant and Wildlife Sciences, Brigham Young University, Provo, UT 84604;; ^d^Centre National de la Recherche Scientifique (CNRS), ECOBIO - UMR 6553, Université de Rennes, 35042 Rennes, France;; ^e^Water Mission Area, US Geological Survey, Menlo Park, CA;; ^f^Department of Hydrogeology, Helmholtz Centre for Environmental Research - Zentrum für Umweltforschung (UFZ), 04318 Leipzig, Germany;; ^g^Division of Hydrologic Modeling, University of Bayreuth, 95447 Bayreuth, Germany;; ^h^Ecole Nationale du Génie Rural, des Eaux et des Forêts (ENGREF), Agroparistech, 75231 Paris, France;; ^i^Department of Hydrology, Bayreuth Center of Ecology and Environmental Research, 95447 Bayreuth, Germany;; ^j^Institut National de la Recherche Agronomique (INRA), Sol Agro et Hydrosystème Spatialisation, UMR 1069, Agrocampus Ouest, 35042 Rennes, France;; ^k^Institut National de Recherche en Sciences et Technologies pour l’Environnement et l’Agriculture (Irstea), RiverLy, Centre de Lyon-Villeurbanne, 69625 Villeurbanne, France

**Keywords:** groundwater, denitrification, reactivity pattern, transit times, reaction times

## Abstract

Biogeochemical reactions occur unevenly in space and time, but this heterogeneity is often simplified as a linear average due to sparse data, especially in subsurface environments where access is limited. For example, little is known about the spatial variability of groundwater denitrification, an important process in removing nitrate originating from agriculture and land use conversion. Information about the rate, arrangement, and extent of denitrification is needed to determine sustainable limits of human activity and to predict recovery time frames. Here, we developed and validated a method for inferring the spatial organization of sequential biogeochemical reactions in an aquifer in France. We applied it to five other aquifers in different geological settings located in the United States and compared results among 44 locations across the six aquifers to assess the generality of reactivity trends. Of the sampling locations, 79% showed pronounced increases of reactivity with depth. This suggests that previous estimates of denitrification have underestimated the capacity of deep aquifers to remove nitrate, while overestimating nitrate removal in shallow flow paths. Oxygen and nitrate reduction likely increases with depth because there is relatively little organic carbon in agricultural soils and because excess nitrate input has depleted solid phase electron donors near the surface. Our findings explain the long-standing conundrum of why apparent reaction rates of oxygen in aquifers are typically smaller than those of nitrate, which is energetically less favorable. This stratified reactivity framework is promising for mapping vertical reactivity trends in aquifers, generating new understanding of subsurface ecosystems and their capacity to remove contaminants.

## Significance

Although groundwater is a critical source of drinking water and irrigation, it has been polluted worldwide by agriculture, industry, and domestic activity. Because assessing groundwater quality and recovery rates is challenging, we developed a method for determining where and how quickly nitrate is removed in aquifers using just a few point measurements of groundwater chemistry. This methodology opens new avenues for characterizing catchment-scale nutrient dynamics, including nitrogen, carbon, and silica, with existing datasets for ecosystems around the globe. Understanding the subsurface structure of reactivity would also improve estimates of recovery time frames for polluted ecosystems and inform sustainable limits for anthropogenic activity.

Humans have exceeded the Earth’s capacity to receive and process nitrogen (1), triggering eutrophication in rivers, lakes, and coastal zones, which imposes billions of dollars of ecological and socioeconomic costs annually (2, 3). However, even if anthropogenic inputs of reactive nitrogen (e.g., ${\text{NH}}_{4}^{+}$, ${\text{NO}}_{3}^{-}$) were stopped today, elevated nitrate $\left({\text{NO}}_{3}^{-}\right)$ concentrations in aquifers could persist for decades to centuries, sustaining eutrophication in rivers, lakes, and estuaries (4–7). The capacity of aquifers to immobilize or remove reactive nitrogen is highly variable within and among aquifers, underlying their functional heterogeneity but complicating evaluation of sustainable limits at medium to large scales. Heterogeneity of reactivity is typical of all ecosystems, meaning that information about the biogeochemical transformation of nutrients (7) and the spatial organization of reactivity is necessary to determine ecosystem functioning (8, 9). There is substantial evidence from aquifers around the globe that ${\text{NO}}_{3}^{-}$ is removed by denitrification or retained in the subsurface because of inorganic and organic electron donors (10–17); however, the spatial distribution of these reactants in aquifers remains almost completely unknown. Consequently, new methods to efficiently characterize the vertical and lateral spatial pattern of subsurface reactivity are urgently needed to determine sustainable limits of anthropogenic activity and predict recovery time frames of polluted ecosystems (18, 19).

Denitrification is the primary removal pathway of reactive nitrogen in aquifers (20), and it occurs when three factors coincide: occurrence of denitrifying microorganisms, presence of anoxic conditions, and availability of electron donors (20–24). Denitrification rates in groundwater can be inferred from ${\text{NO}}_{3}^{-}$ disappearance and ${\text{N}}_{2}$ production during transport along flow paths from aquifer recharge to discharge zones. These overall, or bulk, rates are generally interpreted as apparent reaction rates integrating flow, transport, and reactive conditions over large aquifer widths and depths (24–26). Consequently, the same apparent rates could result from either slow reactions over long flow paths or fast reactions in localized zones. The assumption of continuous reactivity may be appropriate for some sedimentary aquifers, but structured vertical patterns of denitrification activity are likely the norm in most aquifers because both the abundance of microbial communities and the availability of electron donors vary in three dimensions (27). Concerning electron donors, the abundance of organic carbon typically decreases with depth, while availability of reduced iron and pyrite commonly increases with depth in discrete layered formations (23, 28). The decrease in organic carbon is not linear, with the amounts in the soil zone (1–2 m) often being orders of magnitude greater than those in the underlying geological media (29–31).

## Inferring Stratified Reactivity in Groundwater

In complex environments, such as aquifers, the investigation of reactivity requires the deconvolution of information about hydrological mixing, time-varying solute inputs, and reaction rates (7). Tracer concentrations in wells, *C*(*t*)s, can be modeled by weighting the input concentrations, *C*_*0*_(*t*)s, by the transit time distribution, *p*(*u*), using Eq. **1** (25, 32):

$$C\left(t\right)=\int_{0}^{\infty }p\left(u\right)\;C_{0}\left(t-u\right)\;r\left(u\right)\;\;du.$$ [1]

Degradation of ${\text{O}}_{2}$ and ${\text{NO}}_{3}^{-}$ is generically expressed by a reaction term, *r*. The integration variable *u* represents the transit time of a water parcel. Assessing successive ${\text{O}}_{2}$ and ${\text{NO}}_{3}^{-}$ depletion for each well is achieved by deconvoluting the effects of vertically separated flow paths (6, 33). For some of these flow paths, oxic conditions may prevent denitrification, while in others, conditions favorable to denitrification could have resulted in complete depletion of ${\text{NO}}_{3}^{-}$.

We developed a framework to constrain transit times (water travel times from the water table to the sampling point), reaction times (inverses of the first-order rate coefficient), and reaction locations. Because biological reactions are typically fast compared with total groundwater transit times (34, 35), reaction times are primarily controlled by transport and access to electron donors. While this has long been known for reaction times generally, we found that differences in apparent ${\text{O}}_{2}$ and ${\text{NO}}_{3}^{-}$ reaction times can inform about the spatial pattern of reactivity. When electron donors are only available at depth, apparent ${\text{O}}_{2}$ reaction times will be greater than apparent ${\text{NO}}_{3}^{-}$ reaction times, because ${\text{O}}_{2}$ must be sufficiently depleted before ${\text{NO}}_{3}^{-}$ reduction can start. We describe this configuration of deep reactivity as a late start. Conversely, an early stop of reactivity due to a decrease of electron donors with depth results in apparent ${\text{NO}}_{3}^{-}$ reaction times being larger than apparent ${\text{O}}_{2}$ reaction times.

**Fig. 1.**
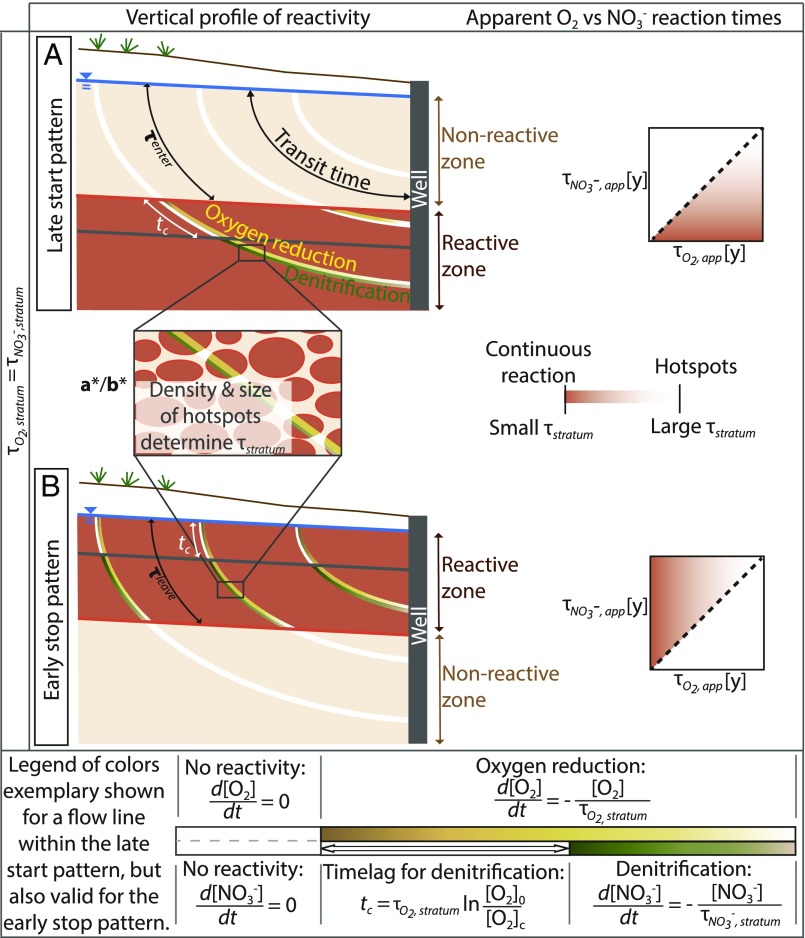
Schematic representation of the stratified reactivity framework. Potential vertical profiles of reactivity in an aquifer (*Left*) and resulting apparent reaction times (*Right*) are shown. The framework assumes similar stratum reaction times, *τ*_*stratum*_, (within a given layer) for O_2_ and NO_3_^−^. A late start or an early stop of reactions along the flow paths results in differences in apparent O_2_ and NO_3_^−^ reaction times. (*A*) Late start of reactivity creates the late start pattern, where the subsequent O_2_ and NO_3_^−^ reduction only starts after a time, *τ*_*enter*_, when the water reaches the reactive layer. The late start increases the apparent O_2_ reaction time compared with the stratum O_2_ reaction time. The subsequent apparent NO_3_^−^ reduction is only marginally affected, because the time for NO_3_^−^ reduction starts only after O_2_ is depleted, resulting in longer observed apparent O_2_ reaction times compared with NO_3_^−^. (*B*) Early stop of reactivity results in NO_3_^−^ degradation first being limited by O_2_ and then by the absence of electron donors. In the early stop scenario, the apparent reaction time for O_2_ is smaller than for NO_3_^−^, and the difference between the two informs the characteristic time, *τ*_*leave*_, when reactive elements leave the reactive stratum. Evenly distributed electron donors throughout the aquifer correspond to a small *τ*_*enter*_ and a large *τ*_*leave*_ and result in a uniform reactive stratum with a sequential reduction of O_2_ and NO_3_^−^ starting at the water table. The relation of apparent O_2_ and NO_3_^−^ reaction times in the case of a uniform reactive stratum is represented by the dashed line in the plot of apparent reaction times (also *SI Appendix*, section S2). The hot spot pattern is compatible with both stratified reactivity patterns (a*/b*).

Fig. 1 shows possible patterns of stratified reactivity that result in different relations of apparent ${\text{O}}_{2}$ and ${\text{NO}}_{3}^{-}$ reaction times. The patterns are determined by vertical differences in availability of electron donors, which either occur primarily in deeper strata because of the depletion of reduced elements by weathering in shallow strata (Fig. 1*A*) or in shallow strata due to abundant surface-derived organic carbon (Fig. 1*B*). We define the stratum reaction time as the inverse of the characteristic first-order rate coefficient for a discrete reactive layer or stratum. Assuming that reaction rates in the reactive stratum are similar for ${\text{O}}_{2}$ and ${\text{NO}}_{3}^{-}$ (36), differences in apparent reaction rates and times for ${\text{O}}_{2}$ and ${\text{NO}}_{3}^{-}$ are only related to the time needed to enter (late start pattern, Fig. 1*A*) and to leave (early stop pattern, Fig. 1*B*) the reactive stratum. Within the reactive stratum, reactivity can be uniform or randomly distributed in microsites (hot spots; a*/b*, Fig. 1). The relative importance of uniform and hot spot reactivity within the stratum can be assessed by the stratum reaction time, with lower density of hot spots corresponding to longer reaction times. This uniform/hot spot ratio can be accounted for with the well-established concept of exposure time scales, which extracts the duration of contact with hot spots along the flow path (7, 37, 38).

We inferred the stratification of reactivity (i.e., the increasing or decreasing availability of electron donors with depth) of a crystalline aquifer in western France by analyzing the relationship between apparent ${\text{O}}_{2}$ and ${\text{NO}}_{3}^{-}$ reaction times for 16 individual wells. Apparent reaction times were derived by using Eq. **1** and environmental tracers (*Materials and Methods*). We synthesized published apparent ${\text{O}}_{2}$ and ${\text{NO}}_{3}^{-}$ reaction times from all studies, to our knowledge, that had appropriate data consisting of first-order reaction times estimated from the deconvolution approach or multiwell sites (i.e., each site contains three or four wells screened at multiple depths) in a transect. This synthesis includes data from 23 individual wells of an aquifer in the Central Valley of California (39) and five multiwell sites from five aquifers located in glacial sediments in Brighton, Michigan (40); Perham, central Minnesota (41); and the Anoka Sand Plain, Minnesota (42), as well as alluvial aquifers on the coastal plain of North Carolina (43) and in the Central Valley of California (24). Published apparent reaction times of the individual wells were also determined by Eq. **1** and environmental tracers, whereas apparent reaction times at the multiwell sites resulted from the relation of measured concentrations to tracer-based groundwater ages based on the piston flow assumption. This method at the multiwell sites allowed independent verification of the stratified reactivity framework because the apparent reaction times of ${\text{O}}_{2}$ and ${\text{NO}}_{3}^{-}$ could be estimated directly with data from multiple depths.

## Results and Discussion

### Observed Vertical Reactivity Patterns and Reaction Times.

**Fig. 2.**
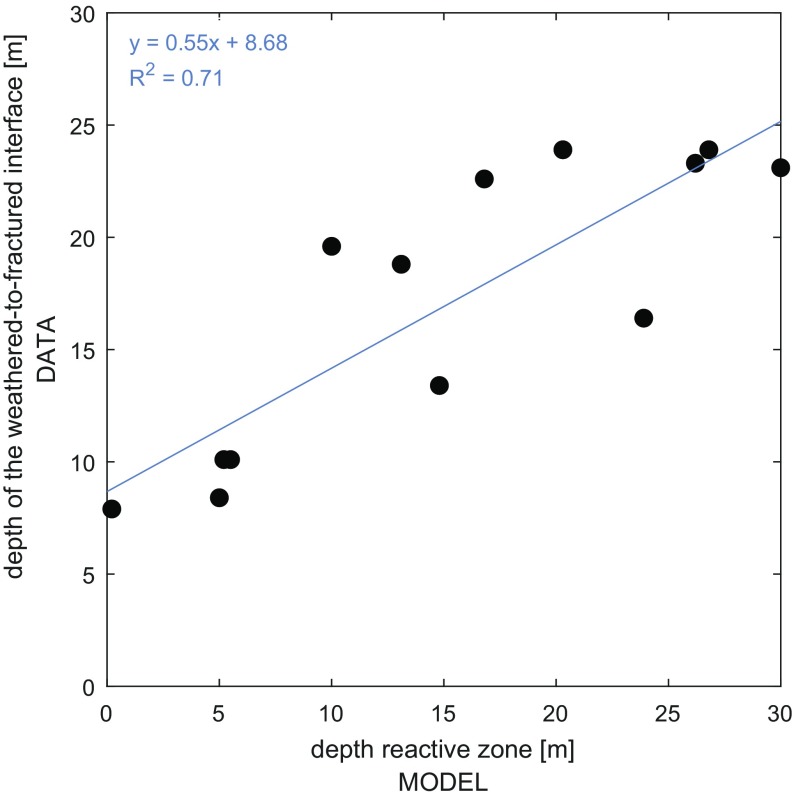
Relationship between the depth of the weathered-to-fractured interface and the depth where the reactive zone is inferred by the stratified reactivity framework. Data of the weathered and fractured interface depth were provided by the French Geological Survey (6). Depths of the reactive zone were determined by examining the depth of a water parcel at the time it enters the reactive zone, *τ*_*enter*_, and calculated via the stratified reactivity framework. The interface of the weathered and fractured zone thickness has been found to be reactive in several crystalline aquifers in Brittany, France (45). This supports the validity of the stratified reactivity framework.

The relations of apparent ${\text{O}}_{2}$ and ${\text{NO}}_{3}^{-}$ reaction times obtained from the unconfined crystalline aquifer located in an agricultural area in Brittany, France (6), show a predominance of the late start pattern with apparent ${\text{O}}_{2}$ reaction times larger than apparent ${\text{NO}}_{3}^{-}$ reaction times (88% of the sampling locations; *SI Appendix*, section S1). The dominance of the late start pattern appears to be driven by biotite and sulfide minerals, which are found in borehole cuttings from deeper fractured zones (44), with reactions occurring between the weathered and fractured zones (44–48). The time to enter the reactive zone, *τ*_*enter*_, is verified by comparing the depth when a water parcel reaches the reactive zone and the depth of the interface between the weathered and fractured zones (Fig. 2). The depths of the reactive zone were determined by the depth of a water parcel at the time it enters the reactive zone by using information about groundwater flow and transit times from a numerical groundwater flow model (6). Alternatively, analytical solutions for the depth versus age relationship (42) could be used to determine the depth of the reactive zone. Existing ${\text{NO}}_{3}^{-}$ stratum reaction times measured in Brittany show a similar range as determined here, ranging within a few hours and several years depending on the electron donor availability (47–51).

**Fig. 3.**
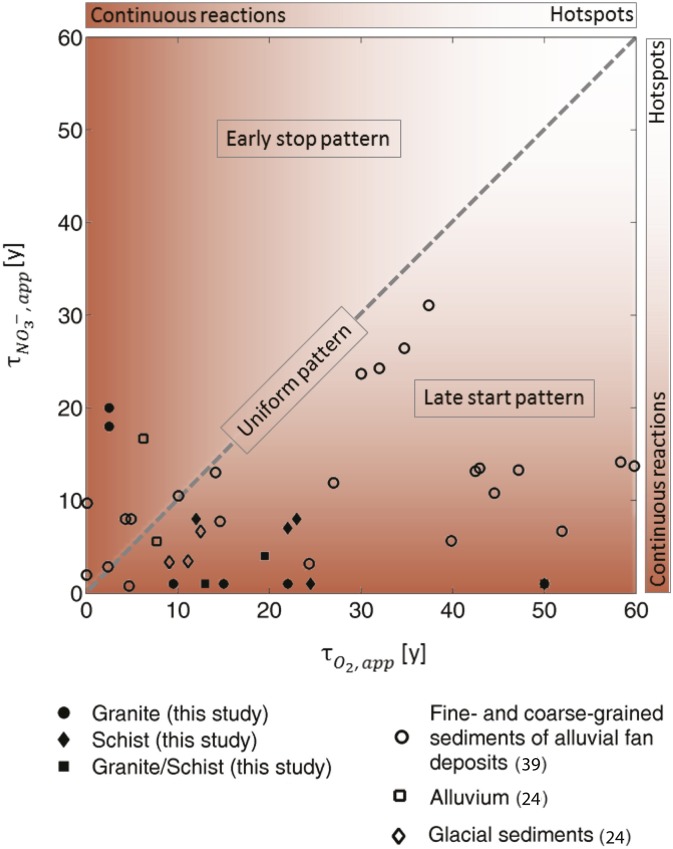
Apparent O_2_ versus apparent NO_3_^−^ reaction times determined from field data for different aquifer types. The comparison provides information on the vertical pattern of reactivity (late start and early stop pattern) and related stratum reaction times. The dark brown color in the background indicates continuous reactions within the reactive stratum with short reaction times, and the light brown background indicates hot spots within the reactive stratum with larger reaction times depending on the density of hot spots (*Materials and Methods* and *SI Appendix*, section S2). The dominance of the late start pattern in aquifers is noticeable, with 79% of the data points indicating that reactions occur after a nonreactive time lag.

The synthesis of new and published data (44 sampling locations in total) shows that only 5% of the observed data points indicated a uniform reactivity pattern (data points along the 1:1 line ± 1 y) independent of aquifer type (e.g., crystalline, glacial, sedimentary; Fig. 3). Approximately 79% of the data points had longer apparent reaction times for ${\text{O}}_{2}$ than for ${\text{NO}}_{3}^{-}$, indicating a late start pattern. This suggests that relatively deep electron donor sources from geological deposits of organic matter or sulfide minerals exert a strong control on the reduction of ${\text{O}}_{2}$ and ${\text{NO}}_{3}^{-}$ independent of the water table and well depth (*SI Appendix*, section S1.3). This is supported by studies of the alluvial aquifer in the Central Valley of California and the glacial sediment aquifers in Minnesota. In these aquifer systems, the solid phase electron donors are the main energy sources for groundwater denitrification compared with surface-derived dissolved organic carbon (DOC) that is not sufficiently available based on electron and mass balance calculations (42, 52). In the alluvial aquifer in California, pyrite and reduced iron minerals (along with organic matter) have been identified as electron donors (52). Increased sulfate concentrations at the sampling location in Brighton, Michigan, suggest that iron sulfides are potential electron donors for reduction reactions. The analysis of apparent reaction times obtained at the multiwell sites (24) allows one to sample the vertical profile of the aquifer and to independently verify the stratified reactivity framework. Apparent reaction times indicate a late start pattern that is supported by detailed vertical profiles (substrata scale) (23, 42) showing the electron donor availability and degradation of ${\text{O}}_{2}$ and ${\text{NO}}_{3}^{-}$ with depth. ${\text{NO}}_{3}^{-}$ can be quickly reduced when it encounters reactive strata shown for aquifers worldwide (47, 53, 54), providing additional evidence on the prevalence of deeper reactivity.

Sixteen percent of the data points showed the early stop pattern, with shorter apparent reaction times for ${\text{O}}_{2}$ than for ${\text{NO}}_{3}^{-}$. For these sites, the predominant electron donor is likely surface-derived DOC that has not been mineralized in the unsaturated zone before reaching the aquifer (23, 24, 29, 55, 56). The low occurrence of the early stop pattern in our sample could be partially due to human activity, because increased nitrogen application and disruption of soil aggregates during cultivation decrease organic carbon in agricultural soils, decreasing DOC, the primary electron donor in surface and near-surface waters (57).

Apparent ${\text{O}}_{2}$ and ${\text{NO}}_{3}^{-}$ reaction times at the studied sites varied widely, indicating both uniform and hot spot dynamics in reactive strata. This diversity of sites with different subsurface characteristics and nutrient loads demonstrates the general utility of the proposed stratified reactivity framework for extracting information about the location and intensity of ${\text{O}}_{2}$ and ${\text{NO}}_{3}^{-}$ reactions from apparent reaction times. Atmospheric tracers and dissolved gases are preferable for deriving apparent reaction times at the stratum scale, because ${\text{NO}}_{3}^{-}$ rate estimates are scale-dependent (25) and approaches such as in-situ mesocosms do not sample the full stratum (52).

One of the central advantages of the stratified reactivity framework is that it relies on widely measured analytical parameters (e.g., solute concentrations, dissolved gas concentrations) and allows identifying the dominant reactivity pattern by analyzing apparent reactivity and not actual rate measurements with an individual sampling location. Used data and common modeling approaches denote the framework as easily deployable to make advanced inferences about the reactivity state of the investigated object. A combination of reactivity patterns is conceivable and would need further investigations. Nevertheless, the stratified reactivity framework could allow regional and interbiome comparison of subsurface reactivity to quantify the relative influence of climate, geology, and surficial processes (e.g., human disturbance, ecosystem development) in determining initial groundwater chemistry and removal capacity. From the perspective of the receiving surface waters, the framework could also analyze downstream river quality data to infer complex ecohydrological processes in headwater catchments, a long goal of freshwater ecology and land management (58, 59).

### Implications of Stratified Reactivity for Water Quality.

The observed differences in apparent ${\text{O}}_{2}$ and ${\text{NO}}_{3}^{-}$ reaction times reveal important vertical patterns in denitrification. Groundwater recovery times from ${\text{NO}}_{3}^{-}$ pollution are related to the heterogeneous flow paths in the subsurface and their intersections with reactive strata (20, 28, 60). Our reanalysis of apparent ${\text{O}}_{2}$ and ${\text{NO}}_{3}^{-}$ reaction times revealed that 79% of groundwater reaction times (Fig. 3) are consistent with the late start pattern, indicating exposure to reactive conditions only after some time spent traveling through a nonreactive zone. This pattern of lithogenic electron donor sources implies relatively conservative transport of ${\text{NO}}_{3}^{-}$ in the upper part of the aquifer. Depending on the depth of the reactive stratum, this means that a considerable amount of ${\text{NO}}_{3}^{-}$ could persist in the upper aquifer, with implications for near-surface groundwater quality. Conversely, deeper reactive stratum can have a higher removal capacity for deeper water resources, although it is clear that this protective capacity can be exceeded (61).

In the case of the early stop pattern observed in 16% of the sites, which occurs when surface-derived electron donors decrease with depth, ${\text{NO}}_{3}^{-}$ could pass through the reactive layer if electron donors are insufficient to substantially decrease ${\text{O}}_{2}$ and ${\text{NO}}_{3}^{-}$ concentrations. Remaining ${\text{NO}}_{3}^{-}$ below the reactive stratum would then persist for long time scales in the deeper aquifer, significantly affecting the quality of water resources and fueling the long-term eutrophication of surface waters as a function of the aquifer contribution to the overall streamflow.

Numerous studies deal with denitrification in aquifers and show that ${\text{NO}}_{3}^{-}$ is potentially removed in the subsurface, but solving the issue of heterogeneity of reactions and locating them have remained extremely challenging. Existing estimates and predictions of denitrification in aquifers often neglect stratified reactivity (55, 62), but accounting for stratified reactivity is central to understanding subsurface denitrification and global nitrogen cycling. Global estimates of groundwater denitrification of ∼44 Tg of N per year derive from models of ${\text{NO}}_{3}^{-}$ reduction in shallow groundwater (0–5 m) with the assumptions that DOC serves as an electron donor and that denitrification is negligible in deeper zones (55, 63, 64). Reconsidering this estimate by accounting for prevalent denitrification activity in deeper strata, the actual nitrate reduction in groundwater might be greater, toward the estimated upper limit of 138 Tg of N per year (55). By adding information about apparent ${\text{O}}_{2}$ reaction times, previously estimated ${\text{NO}}_{3}^{-}$ reduction times could be reanalyzed with the stratified reactivity framework to quantify the reactive front (depth of electron donors) and related ${\text{NO}}_{3}^{-}$ stocks in reactive and nonreactive subsurface zones. The reactive patterns inferred from aquifers in different hydrogeological settings with this framework demonstrate the general applicability and capacity to improve predictions of subsurface reactions that affect groundwater as well as surface water quality. Because this methodology discerns spatial patterns using a relatively small number of samples (compared with well-transect studies) that can be collected from existing wells, it facilitates more widespread assessment of natural attenuation time frames and sustainable loading limits in agricultural landscapes where water quality is rapidly degrading, including in the developing world, where groundwater contamination most directly affects human health (59, 65–67). Finally, while we applied the stratified reactivity framework to explore the denitrification patterns in aquifers, the method is general and could be used to infer reactivity patterns in “black box” situations using apparent reaction times and various chemical compounds, such as silica and carbon. For example, our proposed method could generate understanding about broader climatic, geological, and socioecological controls on weathering, freshwater chemistry, and transport of solutes from land to sea.

## Materials and Methods

### Study Sites.

We used new and published data from 44 well locations from six different aquifers in France and the United States to infer reactivity patterns in groundwater. We investigated 16 well locations of the Pleine-Fougères aquifer in Brittany, France. We used pointwise atmospheric and anthropogenic tracer data [chlorofluorocarbons (CFCs), as well as ${\text{O}}_{2}$, ${\text{NO}}_{3}^{-}$, and ${\text{N}}_{2}$ excess] interpreted within adapted lumped parameter models to derive apparent reaction times (*Modeling Approach* and *SI Appendix*, sections S1.1 and S1.2). The unconfined crystalline aquifer is partly characterized by granite and schist. The 16 wells are distributed over the 76-km^2^ study area with depths ranging from 28 to 98 m below the surface. The water table remains shallow, from close to the surface down to a few meters. The tracer-based mean groundwater age is around 40 y, with mean travel distances around 350 m (6).

We supplemented the dataset with already published ${\text{O}}_{2}$ and ${\text{NO}}_{3}^{-}$ apparent reaction times that were derived using a similar method to the Pleine-Fougères study at 23 wells in another aquifer in the United States. Published data of 23 wells were obtained from the regional alluvial fan aquifer in the Central Valley of California in a 4,000-km^2^ study area (39, 68). The 24 wells’ depths ranged from 3 to 95 m below the water table, with a median of 33 m, and water table depths ranged from 2 to 106 m below ground surface, with a median of 11 m. Tracer-based groundwater ages range from 0.2 to >100 y, with a median of 25 y. The five other ${\text{O}}_{2}$ and ${\text{NO}}_{3}^{-}$ apparent reaction times were obtained from a multiwell analysis, each from a separate aquifer system located in the United States (24). The aquifer in Stevinson, California, is also located in the Central Valley of California containing alluvial sand, silt, and clay, with a saturated thickness of 24 m. The water table is 5–10 m below the ground surface, and tracer-based groundwater ages range from approximately 10 to 30 y (52). The aquifer in Brighton, Michigan, is characterized by glacial outwash and till, with a saturated thickness of 17 m. The unsaturated zone is between 3 and 6 m below the ground surface. The tracer-based groundwater age varies between approximately 1 and 30 y (24, 40). Another aquifer is located in the North Carolina coastal plain. The saturated subsurface contains marine deposits of medium to fine sand and is 13 m thick. Tracer-based groundwater ages are between approximately 3 and 40 y. The water table is shallow, with just a few meters below the ground surface (24, 43). The Perham and Princeton aquifers, located, respectively, in central Minnesota and the Anoka Sand Plain in Minnesota, are characterized by glacial outwash with a saturated thickness of 5 m and 14 m, respectively. The water table depth of the Perham aquifer ranges from close to the ground surface up to a depth of 10 m. Tracer-based groundwater ages range between 5 and 50 y. The Princeton aquifer shows tracer-based groundwater ages within <1 and 30 y, and the water table is between 0 and 4 m depth below the ground surface (24, 41, 42).

Mixing of different waters arriving at the well allows the determination of characteristic ${\text{O}}_{2}$ and ${\text{NO}}_{3}^{-}$ reaction times. Based on apparent reaction times, the time spent in the reactive stratum, the time needed to enter the reactive stratum (late start pattern) or to leave it (early stop pattern), and the characteristic denitrification time in the reactive stratum (stratum reaction time) can be deduced (*SI Appendix*, section S2).

### Definitions.

#### Apparent reaction time.

The apparent reaction time, $\tau_{app}$, derives from the degradation of an element from its inlet concentration in the aquifer, *C*_*0*_(*t*), to its sampled concentration, *C*(*t*). Assuming first-order kinetics and reaction occurrence along the whole flow path in uniform or randomly distributed hot spots, the relative reduction of concentration can be expressed as a function of travel time, *t*:

$$r_{app}\left(t\right)=\exp\left(-\;k_{app}t\right)\mathrm{with}\;k_{app}=\frac{1}{\tau_{app}}.$$ [2]

Sampled concentrations *C*(*t*)s are obtained by using Eq. **1**.

#### Stratum reaction time.

The stratum reaction time, $\tau_{stratum}$, characterizes the degradation of a chemical compound within the reactive stratum of the aquifer, following either the late start or early stop pattern (Fig. 1). The reaction rate along a flow line solely depends on the time spent in the reactive stratum, $t_{stratum}$. Assuming first-order kinetics, the relative stratum reaction rate along a flow line is expressed as

$$\begin{array}{c} r_{stratum}\left(t\right)=\exp\left(-k_{stratum}t_{stratum}\right)\\ \mathrm{with}\;k_{stratum}=\frac{1}{\tau_{stratum}}\\ \begin{array}{c} t_{stratum}=t-\tau_{enter}\;\text{if}\;t>\tau_{enter},\text{otherwise}\;t_{stratum}=0\;\text{for the late start pattern}\\ t_{stratum}=t\;\text{if}\;t<\tau_{leave},\text{otherwise}\;t_{stratum}=\tau_{leave}\;\text{for the early stop pattern}. \end{array} \end{array}$$ [3]

Because the reactive stratum may be determined by lithology, such as for autotrophic denitrification (24), or by surficial processes, such as for heterotrophic denitrification, the vertical stratification of reactivity indicates the type of electron donors supporting denitrification hot spots. The stratum reaction time would thus be substantially larger than the intrinsic reaction time, which can be measured in laboratory experiments with only the reactive minerals.

### Modeling Approach.

A time-based modeling approach was used to infer apparent reaction times and stratum reaction times, as well as the time to enter or leave the reactive stratum at the unconfined crystalline aquifer in Brittany, France. The modeled tracer concentrations at a well, *C*(*t*)s, were calculated from the convolution integral of Eq. **1** (32). CFC dating proxies interpreted within a formerly developed groundwater flow and transport model were used to generate the transit time distributions and CFC concentration distributions, from which we derived mean groundwater ages (6, 69). While site transmissivity and porosity were well constrained by the overall flux and CFC-12 concentrations, local sampling conditions in agricultural wells were calibrated using the detailed conservative tracer information. We evaluated the reliability of measured CFC-12 concentrations by comparing the agreement between all quantified CFCs, sulfur hexafluoride concentrations (another anthropogenic gas used as a tracer of transit time), and dissolved silica concentrations (6, 7, 45, 70). This modeling approach let us include data from wells that were not drilled specifically for scientific monitoring and for which the depths of water arrivals were unknown (26). Alternatively, transit time distributions at well locations could be derived by lumped parameter models (71, 72). The reaction term of Eq. **3** derives from the vertical distribution of electron donors (Fig. 1).

#### Calibration of $NO_{3}^{-}$ inputs to the aquifer.

Land use information from 1991 to 2013 shows a general uniformity of agricultural practices over the catchment area with no significant evolution of specific land use types in any of the well capture zones, supporting the assumption of uniformly distributed ${\text{NO}}_{3}^{-}$ inputs to the aquifer. However, overall ${\text{NO}}_{3}^{-}$ input concentrations have strongly changed, with large increases from 1945 to 1980 and a maximum around 2000, followed by a gradual decline (73). Because of local differences in land management practices, we used the extensive dataset of ${\text{NO}}_{3}^{-}$ concentrations, ${\text{N}}_{2}$ excess, and transit time distributions to reconstruct the ${\text{NO}}_{3}^{-}$ input chronicle ${\left[{\text{NO}}_{3}^{-}\right]}_{0}\left(t\right)$ with Eq. **1**. This method also has the advantage of being applicable in areas where historical contaminant inputs are not known.

#### Calibration of apparent and stratum reaction times.

Apparent and stratum reaction times for ${\text{O}}_{2}$ and ${\text{NO}}_{3}^{-}$ reduction were calibrated to produce the best fit between simulated and measured ${\text{O}}_{2}$ and ${\text{NO}}_{3}^{-}$ concentrations using Eq. **1** for each location. Apparent concentrations are interpreted in terms of apparent ${\text{O}}_{2}$ and ${\text{NO}}_{3}^{-}$ reaction times, and stratum concentrations are interpreted in terms of stratum reaction times and the time to enter or to leave the reactive stratum. The stratum reaction times are similar for ${\text{O}}_{2}$ and ${\text{NO}}_{3}^{-}$ based on similar intrinsic reaction times (36) and similar limitations of the access to the reactive sites in the reactive stratum. We assumed that denitrification only started after ${\text{O}}_{2}$ was depleted below a threshold concentration, ${\left[{\text{O}}_{2}\right]}_{c}$, of 2 mg/L, which creates a time lag for denitrification, *t*_*c*_. Initial ${\text{O}}_{2}$ concentrations, ${\left[{\text{O}}_{2}\right]}_{0}$, of 7 mg/L were considered to be constant over time, agreeing with measured concentrations in shallow piezometers.

## Supplementary Material

Supplementary File
