# Supplementary material for: Stratification of reactivity determines nitrate removal in groundwater
Source: Proc Natl Acad Sci U S A. 2019 Jan 28;116(7):2494–9. doi: 10.1073/pnas.1816892116 (PMC6377467; doi:10.1073/pnas.1816892116)
Supplement: Supplementary File [file pnas.1816892116.sapp.pdf]

Stratification of reactivity determines nitrate removal in groundwater

Tamara Kolbe[a,1,2], Jean-Raynald de Dreuzy[a,b], Benjamin W. Abbott[c,d], Luc Aquilina[a,3], Tristan Babey[a,3], Christopher T. Green[e,3], Jan H. Fleckenstein[f,g,3], Thierry Labasque[a,3], Anniet M. Laverman[d,3], Jean Marçais[h,a,3], Stefan Peiffer[i,3], Zahra Thomas[j,3], Gilles Pinay[d,k]

[a]Centre National de la Recherche Scientifique (CNRS), Géoscience Rennes - UMR 6118, Université de Rennes, 35042 Rennes, France;
[b]Centre National de la Recherche Scientifique (CNRS), Institut National de la Recherche Agronomique (INRA), Observatoire des Sciences de l'Univers de Rennes (OSUR) - UMR 3343, Université de Rennes, 35042 Rennes, France;
[c]Department of Plant and Wildlife Sciences, Brigham Young University, Provo, UT 84604;
[d]Centre National de la Recherche Scientifique (CNRS), ECOBIO - UMR 6553, Université de Rennes, 35042 Rennes, France;
[e]Water Mission Area, US Geological Survey, Menlo Park, CA 94025;
[f]Department of Hydrogeology, Helmholtz Centre for Environmental Research – Zentrum für Umweltforschung (UFZ), 04318 Leipzig, Germany;
[g]Division of Hydrologic Modeling, University of Bayreuth, 95447 Bayreuth, Germany;
[h]Ecole nationale du génie rural, des eaux et des forêts (ENGREF), Agroparistech, 75231 Paris, France;
[i]Department of Hydrology, Bayreuth Center of Ecology and Environmental Research, 95447 Bayreuth, Germany;
[j]Institut National de la Recherche Agronomique (INRA), Sol Agro et Hydrosystème Spatialisation, UMR 1069, Agrocampus Ouest, 35042 Rennes, France; and
[k]Institut National de Recherche en Sciences et Technologies pour l'Environnement et l'Agriculture (Irstea), RiverLy, Centre de Lyon-Villeurbanne, 69625 Villeurbanne, France

[2]Present address: Department of Aquatic Sciences and Assessment, Swedish University of Agricultural Sciences, 75007 Uppsala, Sweden
[3]Authors contributed equally to this work.

Classification: PHYSICAL SCIENCE, Environmental Science

Keywords: groundwater, denitrification, reactivity patterns, transit times, reaction times

[1] To whom correspondence may be addressed. Email: Tamara.Kolbe@posteo.net

www.pnas.org/cgi/doi/10.1073/pnas.1816892116116

S1 Information about the crystalline unconfined aquifer Pleine-Fougères, France

S1.1 Field data

Table S1: CFC-12, O_2, NO_3^- and NO_3^- degraded concentrations for each sampling campaign (Dec 2014, Mar 2015 and Oct 2015)

ID	CFC-12 [pptv]			O_2 [mg/L]			NO_3^-[mg/L]			NO_3^- degraded* [mg/L]		
	Dec-14	Mar-15	Oct-15	Dec-14	Mar-15	Oct-15	Dec-14	Mar-15	Oct-15	Dec-14	Mar-15	Oct-15
1	397.94	251.65	337.34	0.16	0.07	0.54	23.56	26.06	26.57	41.44	32.76	32.73
2	377.06	263.35	313.38	0.44	0.39	0.16	0.00	0.00	0.09	10.44	9.96	8.82
3	521.38	329.29	460.82	5.05	6.18	6.36	51.84	48.98	50.32	12.34	12.63	2.87
4	192.67	107.23	141.06	2.18	1.45	0.71	0.33	0.12	0.00	23.87	35.57	37.88
5	-	227.51	242.92	2.39	2.60	1.63	26.34	33.53	22.78	29.12	22.97	40.43
6	188.08	73.24	108.97	1.54	1.15	1.70	37.76	37.77	37.70	27.58	30.34	27.69
7	-	98.51	99.91	-	0.32	0.29	-	0.00	0.00	-	52.97	46.64
8	437.05	261.75	410.69	6.24	5.59	5.32	63.63	63.57	68.19	19.42	17.13	16.52
9	36.08	36.49	33.63	0.10	0.41	0.20	0.00	0.00	0.00	19.25	17.81	17.54
10	464.76	91.75	33.14	4.16	3.94	0.88	0.00	0.00	0.00	14.17	2.06	7.13
11	11.08	36.36	29.83	0.15	0.85	0.04	0.00	0.00	0.00	10.55	10.20	15.83
12	82.14	126.68	103.18	0.24	1.30	1.60	0.00	0.00	0.04	36.72	44.55	37.18
13	134.58	125.20	95.75	1.56	1.18	0.78	37.96	38.78	24.04	19.52	19.04	19.71
14	435.60	254.18	480.63	6.76	6.78	7.20	42.60	52.37	39.08	2.03	4.11	0.00
15	933.77	-	746.32	2.87	3.88	2.39	32.04	61.81	32.70	11.82	11.45	12.48
16	-	-	220.00	-	-	7.66	-	-	75.02	-	-	0.00

* calculated from dissolved N_2

S1.2 Well locations with information about $\tau_{stratum}$ and τ_{enter}

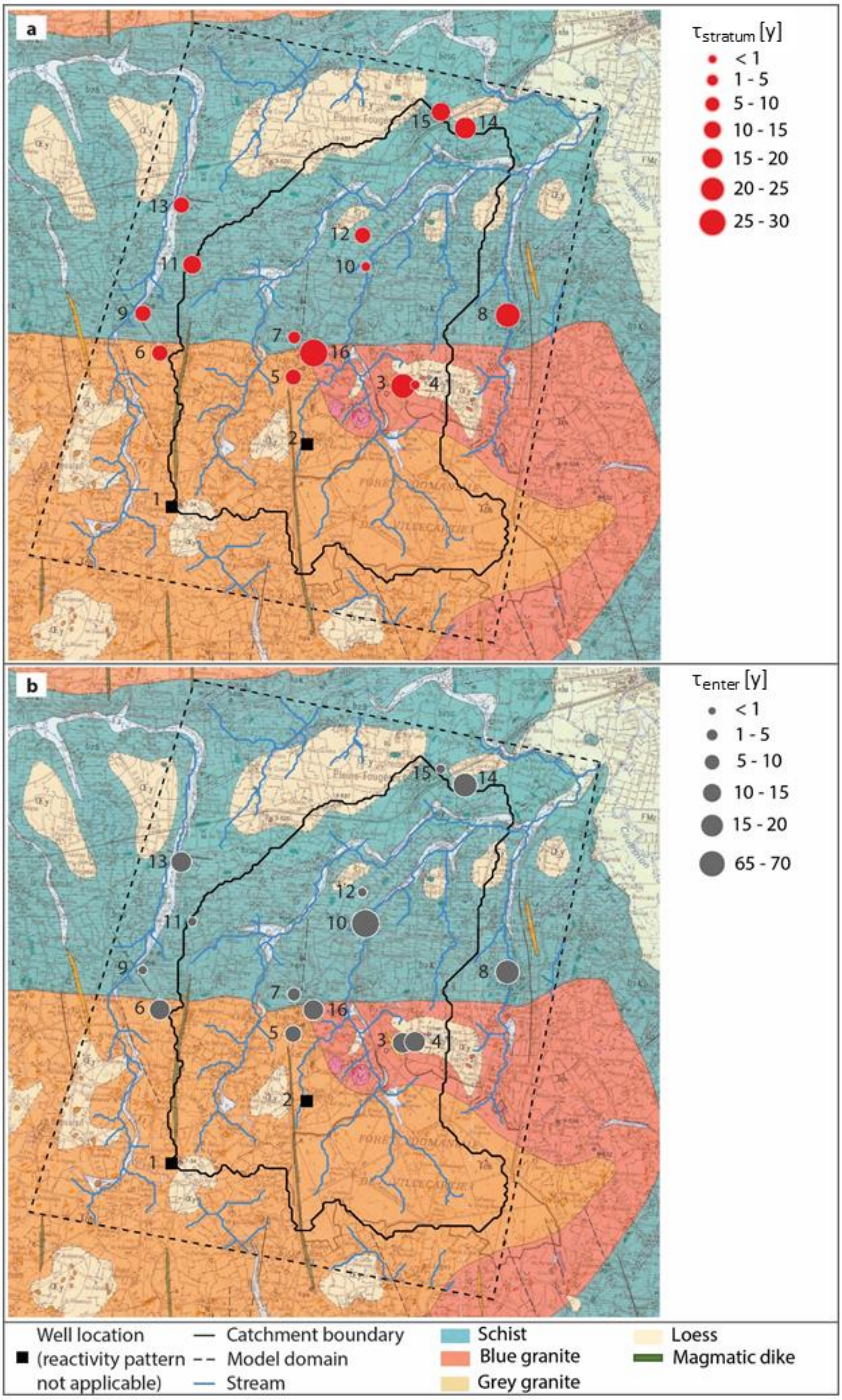

Fig. S1.2: ***a****, represents* $\tau_{stratum}$ *and* ***b****, represents* τ_{enter} *for each well location at the Pleine-Fougères site, France.*

S1.3 Correlation of apparent reaction times with hydrological conditions

Tab. S1.3-1: Hydrological conditions at well locations

ID	Well depth [m]	Water table depth [m]	Thickness saturated zone [m]	CFC based groundwater age [y]	Mean travel distance [m]
1	65	6.4	58.6	34	173
2	70	2.4	67.6	43	436
3	34	11.7	20.3	15	176
4	82	16.3	65.7	45	403
5	94	11.9	82.1	30	422
6	60	20.1	39.9	32	393
7	98	22.7	75.3	43	600
8	80	7.2	75.8	49	765
9	58	1.7	56.3	45	868
10	46	0.1	46	62	1029
11	84	2.4	81.6	40	66
12	34	4.6	29.4	23	173
13	30	1.2	28.8	27	271
14	66	8.3	57.7	21	199
15	28	3.7	24.3	8	122
16	35	20.1	14.9	16	183

Tab. S1.3-2: Pearson product-moment correlation between well characteristics and apparent reaction times

	$\tau_{O_2,app}$	$\tau_{NO_3^-,app}$
Well depth	-0.31	0.01
Water table depth	0.04	-0.45
Thickness saturated zone	-0.33	0.15
CFC based groundwater age	-0.08	0.05
Mean travel distance	0.17	-0.24

S2 Concentrations within the apparent and strata framework – synthetic test case

Here, a uniform transit time distribution and constant input concentrations are used to illustrate the relations between O_2 and NO_3^- concentrations, the stratum reaction times $\tau_{stratum}$ and the apparent reaction times τ_{app}. The uniform transit time distribution is defined between t_{min} = 0 y and t_{max} = 70 y. O_2 and NO_3^- input concentrations are constant over time, e.g. $[O_2]_0$= 7 mg/L and $[NO_3^-]_0$ = 28 mg/L. Relations between apparent and stratum reaction times derive from the equality of apparent and strata concentrations:

$$[O_2]_{app} = [O_2]_{stratum} \quad [S1]$$

$$[NO_3^-]_{app} = [NO_3^-]_{stratum} \quad [S2]$$

S2.1 Concentrations within the apparent framework

Apparent concentrations $[O_2]_{app}$ and $[NO_3^-]_{app}$ in a well are calculated by convoluting their input concentrations $[O_2]_0(t)$ and $[NO_3^-]_0(t)$ with the transit time distribution $p(t)$ and applying a first-order reaction term using Eq. S3 and S4. Reactions are assumed to occur uniformly along the flow line. Expressions are given hereafter in cases where both, O_2 and NO_3^- concentrations, are fully degraded within the range of the transit time distribution, i.e. when the critical time $t_{c,app} = -\tau_{app} \ln\left(\frac{[O_2]_c}{[O_2]_0}\right)$ is within the limits of the time distribution $\left(t_{min} < t_{app,c} < t_{max}\right)$:

$$[O_2]_{app} = [O_2]_0 \frac{\tau_{O_2,app}\left(e^{-\frac{t_{min}}{\tau_{O_2,app}}} - e^{-\frac{t_{max}}{\tau_{O_2,app}}}\right)}{t_{max} - t_{min}} \quad [S3]$$

$$[\mathrm{NO_3^-}]_{app} = [\mathrm{NO_3^-}]_0 \frac{\tau_{NO_3^-,app}\left(1-e^{-\frac{t_{max}-t_{c,app}}{\tau_{NO_3^-,app}}}\right)-t_{min}+t_{c,app}}{t_{max}-t_{min}} \quad \text{[S4]}$$

S2.2 Concentrations in the strata framework – The late start pattern

Within the late start pattern, O_2 and NO_3^- concentrations are calculated by using Eq. S5 and S6. Solutions are given for $t_{min} < \tau_{enter} + t_{c,stratum} < t_{max}$ with $t_{c,stratum} = -\tau_{stratum} \ln\left(\frac{[O_2]_c}{[O_2]_0}\right)$ when both, O_2 and NO_3^- reduction, can occur and are not complete:

$$[\mathrm{O_2}]_{stratum} = [\mathrm{O_2}]_0 \frac{\tau_{O_2,stratum}\left(1-e^{-\frac{t_{max}-\tau_{enter}}{\tau_{O_2,stratum}}}\right)+\tau_{enter}-t_{min}}{t_{max}-t_{min}} \quad \text{[S5]}$$

$$[\mathrm{NO_3^-}]_{stratum} = [\mathrm{NO_3^-}]_0 \frac{\tau_{NO_3^-,stratum}\left(1-e^{-\frac{t_{max}-\tau_{enter}-t_{c,stratum}}{\tau_{NO_3^-,stratum}}}\right)+\tau_{enter}+t_{c,stratum}-t_{min}}{t_{max}-t_{min}} \quad \text{[S6]}$$

S2.3 Relations between apparent and stratum reaction times – The late start pattern

Apparent and stratum reaction times are related to yield identical O_2 and NO_3^- concentrations using Eq. S.1 and S.2. Observed O_2 and NO_3^- concentrations can be interpreted within the apparent and strata framework. For example, sampled concentrations $[O_2]$ = 1.5 mg/L and $[NO_3^-]$ = 8.6 mg/L lead to significantly faster apparent denitrification ($\tau_{NO_3^-,stratum}$ = 2.3 y) than apparent O_2 reduction ($\tau_{O_2,stratum}$ = 15.2 y) (Fig. S2.1a). This goes against common ecological sense. The same concentrations interpreted within the strata framework lead to equal stratum reaction time for O_2 and NO_3^- reduction $\tau_{stratum}$ = 5 y and a delay of the reactions τ_{enter} = 10 y, showing a late start pattern (Fig. S2.1b). Within the strata framework, O_2 reduction starts later and occurs faster than within the apparent framework. NO_3^- reduction starts earlier and is slower.

τ_{enter} and $\tau_{stratum}$ can be determined for any couple of $\tau_{O_2,app}$ and $\tau_{NO_3^-,app}$ as long as $\tau_{O_2,app} > \tau_{NO_3^-,app}$. Divergences between apparent and stratum reaction times occur when τ_{enter} progressively increases as revealed by differences in reaction times of O_2 and NO_3^- reduction (Fig. S2.2a). Apparent reaction times logically increase with the stratum reaction time (Fig. S2.2b).

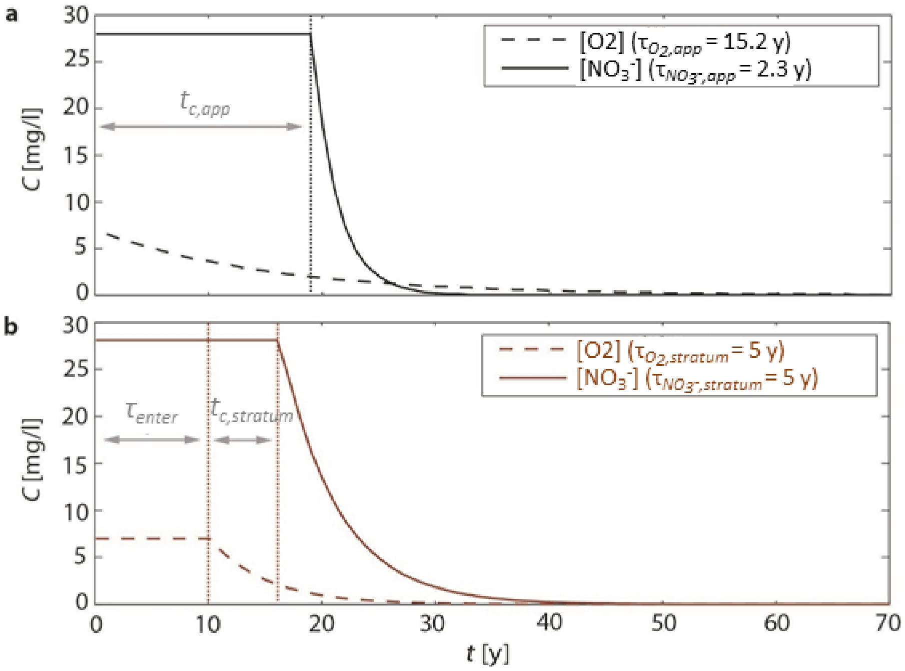

*Fig. S2.1: The evolution of O_2 and NO_3^- concentrations as functions of transit times for a sampling zone. The transit time distribution is uniform. The interpreted concentrations of O_2 and NO_3^- are equal between **a**, the apparent and **b**, the late start pattern.*

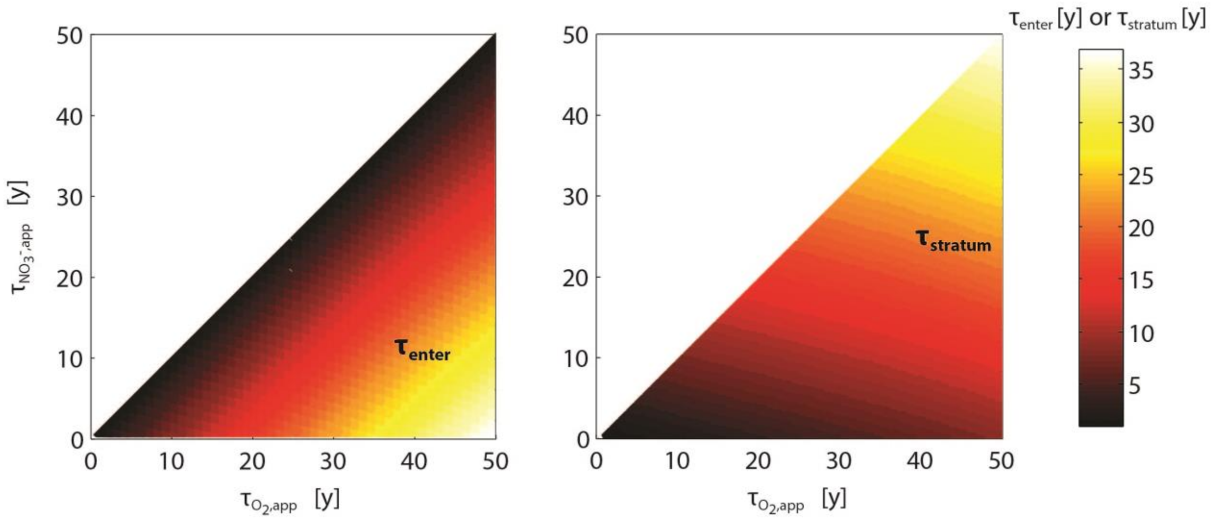

Fig. S2.2: ***a**, τ_{enter} and **b**, $\tau_{stratum}$ as functions of apparent reaction times.*

S2.4 Concentrations in the strata framework – The early stop pattern

Within the early stop pattern, strata O_2 and NO_3^- concentrations are calculated with Eq. S7 and S8. Solutions are valid within the integration limits $t_{min} < \tau_{leave} + t_{c,stratum} < t_{max}$.

$$[O_2]_{stratum} = [O_2]_0 \frac{\tau_{O_2,stratum}\, e^{-\frac{t_{min}}{\tau_{O_2,stratum}}} + \left(t_{max}-\tau_{leave}-\tau_{O_2,stratum}\right) e^{-\frac{\tau_{leave}}{\tau_{O_2,stratum}}}}{t_{max}-t_{min}} \qquad [S7]$$

$$[NO_3^-]_{stratum} = [NO_3^-]_0 \frac{\tau_{NO_3^-,stratum} + \left(t_{max}-\tau_{leave}-\tau_{NO_3^-,stratum}\right) e^{-\frac{\tau_{leave}-t_{c,stratum}}{\tau_{NO_3^-,stratum}}} + t_{c,stratum}-t_{min}}{t_{max}-t_{min}} \qquad [S8]$$

S2.5 Relation between apparent and stratum reaction times – The early stop pattern

Larger apparent reaction times for NO_3^- than for O_2 are representative for the early stop pattern and show incomplete reactions, which can be interpreted as long as the NO_3^- and O_2 apparent times do no diverge too strongly.

Fig. S2.5 demonstrates how τ_{leave} and $\tau_{stratum}$ evolve in the plot of apparent O_2 and NO_3^- reaction times. With a large time to leave the reactive zone, the relation of apparent reaction times approaches the 1:1 line, showing a similar pattern as for uniform distributed reactivity (Fig. S2.5a). Within the early stop pattern, apparent O_2 reaction times are strongly impacted by stratum reaction times (Fig. S2.5b).

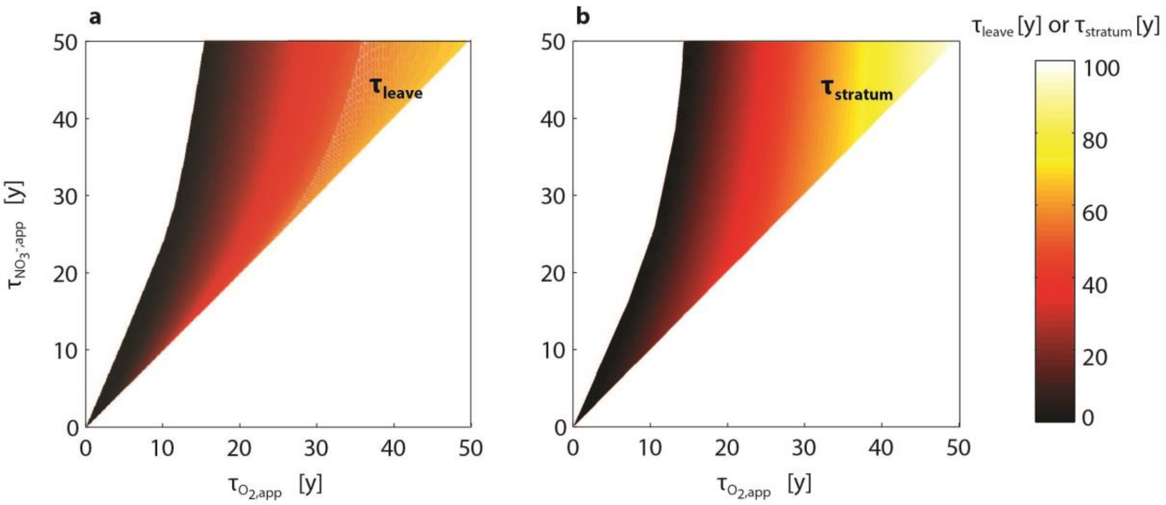

*Fig. S2.5: Evolution of **a**, τ_{leave} and **b**, $\tau_{stratum}$ within the plot of apparent reaction times.*
